# Surface Analysis of Coal Indicating Neutral Red Enhances the Precursor Steps of Methanogenesis

**DOI:** 10.3389/fmicb.2020.586917

**Published:** 2020-11-09

**Authors:** Priyanka Srivastava, Christopher Marjo, Alireza Gerami, Zackary Jones, Sheik Rahman

**Affiliations:** ^1^School of Chemical Engineering, University of New South Wales, Sydney, NSW, Australia; ^2^Mark Wainwright Analytical Centre, University of New South Wales, Sydney, NSW, Australia; ^3^School of Minerals and Mining, University of New South Wales, Sydney, NSW, Australia

**Keywords:** coal, neutral red, redox mediators, acetogens, methanogenesis, attenuated total reflectance-fourier transform infrared, X-ray photoelectron spectroscopy

## Abstract

Artificially stimulated, high-yield microbial production of methane from coal is a challenging problem that continues to generate research interest. Decomposition of organic matter and production of methane from coal are the results of multiple redox reactions carried out by different communities of bacteria and archaea. Recent work by our group (Beckmann et al., 2015) demonstrated that the presence of the redox-mediating molecule neutral red, in its crystalline form on a coal surface, can increase methane production. However, hydrolysis and the acetogenesis of the coal surface are essential precursor steps for methane production by archaea. Acetogenesis is the preparation phase of methanogenesis because methanogens can only assimilate acetate, CO_2_ and H_2_ among the products formed during this process. In the present study, the surface chemical analysis of neutral red treated coal using attenuated total reflectance-fourier transform infrared (ATR-FTIR) spectroscopy and X-ray photoelectron spectroscopy (XPS) demonstrate that the acetate production and resulting oxidation of the coal only occurred at few nanometers into the coal surface (at the nanoscale <5 nm). We observed that in the presence of neutral red and groundwater microbes, acetate signals in coal surface chemistry increased. This is the first evidence suggesting that neutral red enhances the biological conversion of coal into acetate. Microscopy demonstrated that neutral red crystals were co-localize with cells at the surface of coal in groundwater. This is consistent with neutral red crystals serving as a redox hub, concentrating and distributing reducing equivalents amongst the microbial community. In this study, the chemical changes of neutral red treated coal indicated that neutral red doubles the concentration of acetate over the control (coal without neutral red), emphasizing the importance of maximizing the fracture surface coverage of this redox mediator. Overall, results suggested that, neutral red not only can benefit acetoclastic methanogens, but also the fermentative and acetogenic bacteria involved in generating acetate.

## Introduction

**FIGURE 1:**
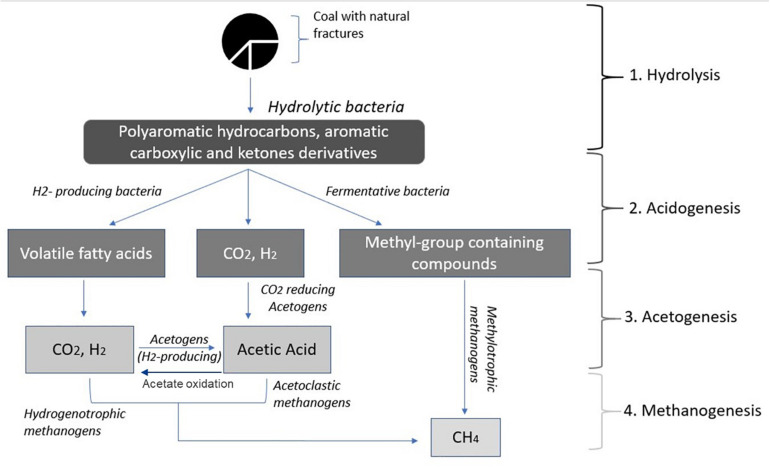
Conversion of coal into methane (adapted from Stra̧poć et al., 2008).

Capturing methane from coal is a growing industry that involves naturally produced methane extraction from coal seam beds. Conversion of coal into methane (Hofrichter and Fakoussa, 2001) involves four different steps; (1) biofragmentation of coal to molecules of lower molecular mass, (2) anaerobic oxidation of biofragmented products to produce volatile fatty acids including acetate, methylated compounds, CO_2_ and H_2_, (3) conversion of volatile fatty acids into CO_2_, H_2_ and acetate, and, (4) conversion of acetate, methylated compounds or H_2_/CO_2_ to methane (Figure 1; Szeinbaum, 2009).

Hydrolytic bacteria involved in step 1, decompose and convert complex organic compounds of coal into polyaromatic hydrocarbons, aromatic carboxylate and ketone derivatives, single-ring aromatics and long-chain alkanes include *Thauera, Azonexus, Geobacter, Acidovorax, Acinetobacter*, and *Pseudomonas*. In step 2, Actinobacteria, Firmicutes, Bacteroidetes, and Spirochetes, are responsible for the fermentation of hydrolyzed substrate to produce CO_2_ and H_2_. In step 3, homoacetogenic bacteria convert carbon dioxide (CO_2_) and molecular hydrogen (H_2_) into acetyl-CoA, through the reductive acetyl-coenzyme A (acetyl-CoA) pathway, also known as the Wood–Ljungdahl pathway. Homoacetogens associated with coal seams include *Acetobacterium, Treponema, Desulfobacterium, Syntrophomonas, Clostridium, Eubacterium, Desulfuromonas, Syntrophobacter, Acetonema, Desulfobacter, Syntrophothermus, Pelobacter, Syntrophobacter*, and *Desulfovibrio*. In step 4, methanogenic archaea utilize acetate, methylated compounds or H_2_ as energy sources and generate methane as a bi-product. Most methanogens in coal seam groundwater carry out acetoclastic or hydrogenotrophic methanogenesis, such as *Methanosarcina, Methanolobus, Methanobacteria, Methanocorpusculum, Methanosaeta, Methanococci, Methanoculleus, and Methanoregula.*

The use of electron mediators to transport reducing equivalents to or from microorganisms has received considerable attention due to their potential to accelerate energy metabolism for growth and to alter the terminal metabolite production toward profitable products (for example: L-glutamate) during the process of fermentation (Nerdahl and Weimer, 2015). The delivery of electrons to fermentative bacteria for biocommodity production at the lab-scale has been successfully implemented using several different mediators including neutral red, benzyl viologen, methyl viologen and humic substances such as anthraquinone 2,6-disulfonate (Hongo and Iwahara, 1979; Park et al., 1999; Clark et al., 2012). Among these, neutral red has shown promising results due to its low toxicity and low standard reduction potential (Eo = −375 mV) (Hongo and Iwahara, 1979).

**FIGURE 2:**
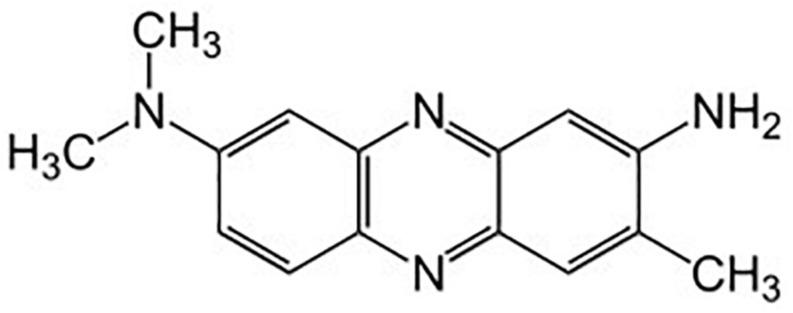
Neutral red.

Neutral red (3-amino-7-dimethylamino-2-methylphenazine, Figure 2) is an amino derivative of the heterocyclic phenazine. Phenazine derivatives have highly reversible redox behavior and act as reducing equivalents in transfer mediating systems (Laviron and Roullier, 1983; Mugnier et al., 1991). Neutral red is a cationic dye and has been used as a histological stain because of its optical sensitivity to pH in the relevant range of pH 6.0–8.0 (Halliday and Matthews, 1983). The molecular structure of neutral red differs under different pH and redox potentials. These structures include NRH^+^, reduced neutral red (NRH_2_) and leuco neutral red (NR). Electrochemical reduction of neutral red yields a yellow solution with a green fluorescence (Park et al., 1999). Reduction of NRH^+^ and NR occurs via two-electron transfer (Azariah et al., 1998). NRH^+^ is reduced to NRH_2_ by a number of bacterial species (Makgill, 1901; Gage and Phelps, 1902; Chamot and Sherwood, 1915). Neutral red has been observed to crystallize as a second crystalline form (a polymorph) (Labine-Romain et al., 2017) at higher temperatures (40–80°C, depending upon the depth of the coal reserves) that may occur in coal seams.

In a previous study by Beckmann in 2015, neutral red crystals (NRCs) were found to increase acetoclastic methanogenesis with coal as a substrate (last step in the conversion of coal to methane), by increasing the phenazine pool in the membrane of methanogens. Whilst methane production was stimulated there was no evidence presented that coal bioconversion increased as would be expected. The aim of this study therefore was to, find out the changes in the surface chemistry of coal in the presence of neutral red and coal seam associated microbes using FTIR and XPS.

Coal samples were incubated under anaerobic conditions in groundwater with its incumbent microbial community in the presence and absence of neutral red for 6 months. Coal samples were then subject to imaging and surface chemical analysis. Imaging revealed the formation of a fine mesh of crystalline neutral red associated with the surface of coal with embedded cells. Acetogenesis was detected by XPS (surface sensitivity ca. 5 nm) and shown to be stimulated by neutral red though these changes were not detected by ATR-FTIR (surface sensitivity ca. 3 micrometers) confirming that acetogenesis was a nanoscale surface phenomenon.

## Materials and Methods

### Anaerobic Enrichments

The coal seam groundwater and coal samples used in this study were collected from different locations. The coal seam groundwater was collected from the Surat Basin, Queensland, Australia, under anaerobic conditions and transported to the laboratory at room temperature. Groundwater was used as an inoculum within 48 h after receipt in the laboratory. Sub-bituminous type coal samples were collected from the No. 7 Lithgow coal seam in the Western Coal Fields of New South Wales, Australia and the Jharia Coal Field, Jharkhand, India. Coal samples were stored dry in the dark under aerobic conditions.

To study the effects of neutral red and microbes on coal surface chemistry, coal samples were incubated with coal seam groundwater (with the incumbent microbial community) or mineral salts (Black-Sea) medium for 6 months under anaerobic conditions in the presence/absence of 250 μM neutral red. Small coal pieces (ca. 1 cm^3^, 5 g total weight per flask) with 80 mL of aqueous phase were added to 120 mL serum bottles with a headspace maintained under an anaerobic atmosphere of N_2_/CO_2_ (80:20). Serum bottles were sealed with butyl rubber stoppers and crimped with 20 mm aluminum caps. The medium used was Black-sea medium and prepared according to Widdel and Pfennig (1981). Neutral red (Sigma) stock solutions (20 mM) were prepared in milliQ water and stored at 4°C. Calculated amount of 20 mM of NR stock solution was added in 80 mL of medium to a final concentration of 250 μM NR, via syringe to maintain the anaerobic conditions (Beckmann et al., 2015). A sodium azide treated sterile control with coal in Black sea media was included. All treatments were prepared in triplicate and incubated static in the dark at 30°C for 6 months.

### Quantification of Acetate and Methane Concentration

Acetate was esterified into ethyl-esters and analyzed by GC-FID. Media samples (500 μl) were added to 200 μl of 100% ethanol, in a 10 ml gas chromatography vial (Agilent). Sulfuric acid (500 μl) was added in a fume hood and the vial was immediately sealed with PTFE/butyl septa attached to an aluminium screw cap (Agilent). Vials were incubated for 45 min at 60°C. Vials were agitated for 5 min at 80°C at 500 rpm on an AOC-5000 Plus auto-sampler before 100 μl headspace samples were injected into a Shimadzu GC-2010 Plus with Flame ionization detector (GC-FID). A DB-FFAP column (30 m × 0.32 mm × 0.25 mm) (Agilent, Santa Clara, United States) was used. The temperature of the column remained constant at 60°C for 6 min.

Methane was also monitored monthly in 100 μL head-space samples by gas chromatography using a Shimadzu GC-2010 with Flame ionization detection (GC-FID) and a Gas-Pro PLOT column (60 m × 0.32 mm, Agilent Technologies, Australia) (Lee et al., 2012). The carrier gas was helium (3 ml min^–1^), and the inlet temperature was 250°C. The oven temperature was held at 100°C for 30 min. Gas samples (100 μl) were taken from the headspace of the serum bottles using a lockable, gas-tight glass syringe (SGE analytical science), and injected into the GC. The methane concentration was quantified by comparison of the peak area of the unknown samples with the four- point standard calibration curve (0–68 μM).

### DNA Extraction and Illumina Sequencing

Microbial communities present in coal seam water were profiled by DNA extraction from 5 mL of coal seam water using a phenol-chloroform extraction method (Penner et al., 2010). Extracted DNA was washed with 80% (v/v) ethanol and dissolved in 40 μL of molecular biology grade water. DNA quality was assessed by gel electrophoresis and quantified using RiboGreen (Qubit Assay Kit, Invitrogen), according to the manufacturer’s instruction. Extracted DNA was used as template DNA for Illumina sequencing. Primers used in PCR to target both bacteria and archaea were 515F: 5′ GTGYCA GCMGCCGCGGTAA 3′ and 806R: 5′ GGACTACNVGGGTWTCTA AT 3′ covering the V4-region of SSU rRNA genes. PCRs contained 1 μl of each primer (20 μM), 20 μl of PCR master-mix (Econo-Taq PLUS GREEN 2X Master Mix), 2 μl of the template DNA, and 17.2 μl of molecular water. The amplification conditions were as follows: initial denaturation at 94°C for 3 min, followed by 35 cycles consisting of denaturation (94°C for 45 s), annealing (1 min at 50°C) and extension (72°C for 90 s), with a final elongation at 72°C for 10 min. The amplified PCR products, were submitted to the “Next generation sequencing facility,” Hawkesbury Institute of Environment, Western Sydney University, Australia, for the Illumina sequencing. QIIME 2 VIEW software were used to analyze the Illumina sequencing results.

### Electron Microscopy

Backscattered-Electron (BSE) imaging was applied to generate high resolution images of the coal surface in the presence of neutral red (David and Ryan, 2013; Spain and Mclin, 2013). In this method, no sample preparation was needed. Coal samples were taken out of the microcosms using pre-sterilized forceps and directly bombarded with electron beam with acceleration of 15 kV. SEM was used to visualize bacteria on coal surfaces. Coal samples were taken out from the microcosms and fixation, dehydration and drying methods were conducted (Fratesi et al., 2004). Cells on the coal surface were fixed by 2.5% of glutaraldehyde in 0.1M Sodium cacodylate buffer and incubated overnight at 4°C. This step was repeated. The old fixative solution was replaced and 2.5% of glutaraldehyde in 0.1M Sodium cacodylate buffer was added. The samples were heated in the microwave for 1 min, to enhance the fixation and dehydration of the coal sample. The samples were quickly washed with 0.1 M sodium cacodylate buffer, and again washed with 1% osmium tetroxide solution, prepared in 0.1M sodium cacodylate buffer. The samples were microwaved for 10 min, and washed with 0.1M of sodium cacodylate solution, followed by washing with distilled water. After washing, samples were dipped into 100% ethanol. Then samples were transferred into 1:2 solution of HMDS (hexamethyldisilazane): 100% ethanol for 20 min. In the final step, the samples were transferred into 100% HMDS solution, and incubated for overnight. The HDMS was completely evaporated and samples were subjected for the sputter coating of platinum. Samples were visualized under SEM. All the steps in this process, were carried out in the fume-hood, because mostly solutions used in the sample preparation are toxic. Using micro-wave bio-wave is more convenient for the fixation and dehydration steps in the HMDS (hexamethyldisilazane) protocol (Hazrin-Chong and Manefield, 2012). At the end of the dehydration process, HMDS was evaporated and the samples were coated with platinum for SEM imaging.

### Surface Analysis of Coal

To characterize chemical modifications on coal surfaces samples were dried in an oven (40°C) to remove water, which interferes with FTIR, and prevents the XPS from achieving operational vacuum. The XPS and ATR-FTIR work was carried out at the Mark Wainwright Analytical Centre at the University of New South Wales, Australia.

#### Attenuated Total Reflection-Fourier Transform Infrared Spectroscopy (ATR-FTIR) Analysis

Attenuated total reflectance-fourier transform infrared was performed to investigate possible changes in coal surface chemistry in the presence of neutral red and a coal groundwater microbial community. Coal samples were analyzed on a Spectrum 100 FTIR spectrometer (PerkinElmer, United States) fitted with a DTGS detector and a diamond/zinc selenide ATR crystal. Samples were measured between 650 and 4000 cm^–1^ for 100 accumulations at randomly selected 1 mm diameter regions by carefully applying pressure to ensure contact with the crystal. Peak fitting of each spectrum was performed to identify any new carbonyl stretching peaks as shoulders overlapping the original C = C stretching peaks and was performed using the GRAMS software (Version 3.03 Thermo Scientific, United Kingdom).

#### X-Ray Photoelectron Spectroscopy (XPS) Analysis

Coal samples were placed in the instrument sample transfer chamber overnight to remove volatiles that inhibit sensitivity. XPS was performed on an ESCALAB250Xi (Thermo Scientific, United Kingdom) using a 500 μm diameter beam of monochromatic Al Kα radiation (photon energy = 1486.6 eV) at a pass energy of 20 eV. The core level binding energies (BEs) were aligned with respect to the C 1 s BE of 284.8 eV. Photoelectron spectra were peak fitted using Advantage software (Version 3.03 Thermo Scientific, United Kingdom).

## Results

### Community Analysis of Coal Seam Groundwater

To examine the impact of neutral red on coal surface chemistry in the presence of a coal seam groundwater microbial community (Surat Basin, Australia), two distinct sub-bituminous coal samples (Jharia, India and Lithgow, Australia) were incubated with neutral red under anaerobic conditions in triplicate for 6 months. Over the incubation period, methane quantification revealed the community was not producing methane, though acetate production was detected (approximately 20–25 μM in the absence of neutral red and approximately 40–45 μM in the presence of neutral red). Sterile controls generated negligible acetate indicating preformed acetate was not desorbing from the coal.

**FIGURE 3:**
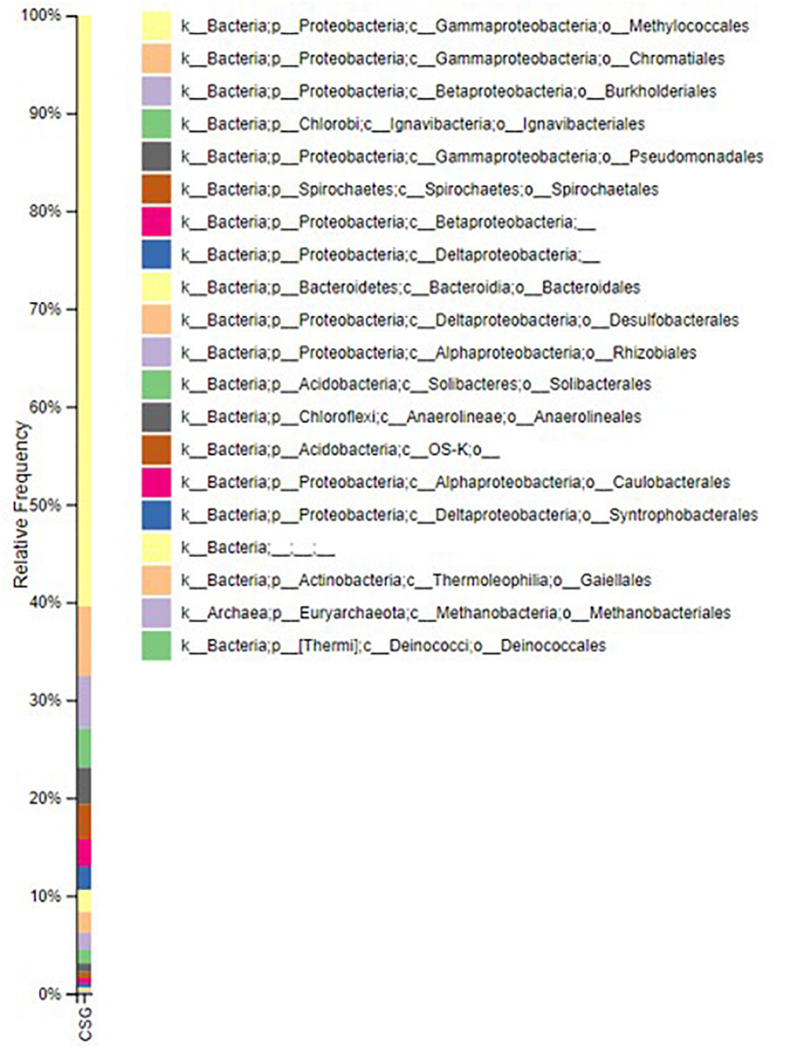
Relative abundance of microbial communities present in the groundwater. The image is generated by using QIIME 2 VIEW software.

Figure 3 shows the composition of the microbial community incumbent in the groundwater used in the experiment based on the V4 region of 16S rRNA gene amplicons. The Illumina data describes the bacterial community composition of the pre-culture inoculum. The community is dominated by a *Methylococcus*. Methanogens (Methanobacteriales) were in low abundance (methane generation graph is provided in the Supplementary Figure 2). Fermentative lineages associated with Chromatiales, Burkholderiales, Spirochetes, Ignavibacteriales, Desulfobacterales, Pseudomonadales, Rhizobiales, Anaerolineales, Chloroflexi, Actinobacteria and Deinococcales with unclassified Betaproteobacteria, Deltaproteobacteria, and Acidobacteria were found. The acetogenic lineages Spirochetales, Desulfobacterales, Chromatiales, and Pseudomonadales, were also amongst the most abundant lineages observed. The sequencing data has been submitted in European Nucleotide Archive. The accession number for the sequence is PRJEB39572.

### Acetate Quantification

**FIGURE 4:**
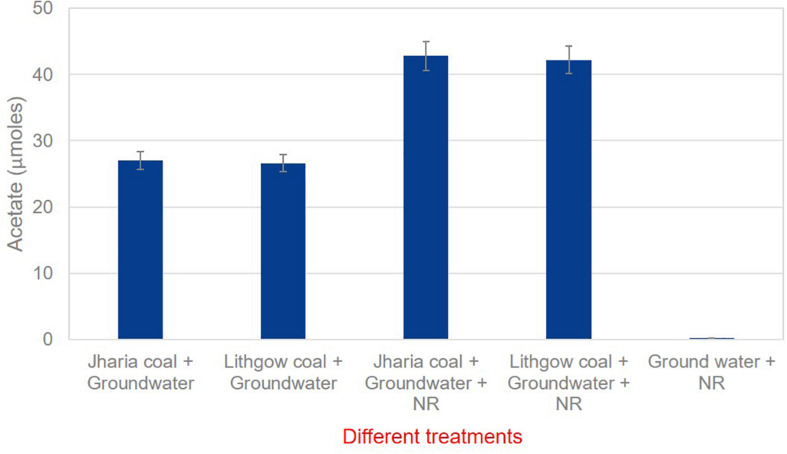
GC data showing the acetate production in the presence and absence of neutral red with Jharia coal and Lithgow coal.

The cultured medium, incubated with coal blocks, neutral red and groundwater, was utilized to find out the amount of acetate produced by the microbial community. Culture medium was taken from each microcosm and analyzed for acetate production from coal by the microbial communities’ present in the groundwater. It was found that 250 μM neutral red has doubled the acetate production in comparison to the controls (no neutral red) (Figure 4). These results suggest that 250 μM of neutral red can enhance the bio-conversion of coal into acetate. The addition of 250 μM neutral red to anaerobic coal fed microbial communities increased the acetate production and generated 42.7 μmoles of acetate from Jharia coal and 42.19 μmoles of acetate from Lithgow coal. However, 27.0 μmoles and 26.61 μmoles of acetate was produced from Jharia coal and Lithgow coal in the absence of NR. In another control, where no coal was provided in the treatment, only neutral red and ground water was provided, 0.2 μmoles of acetate was found.

### Microscopy

**FIGURE 5:**
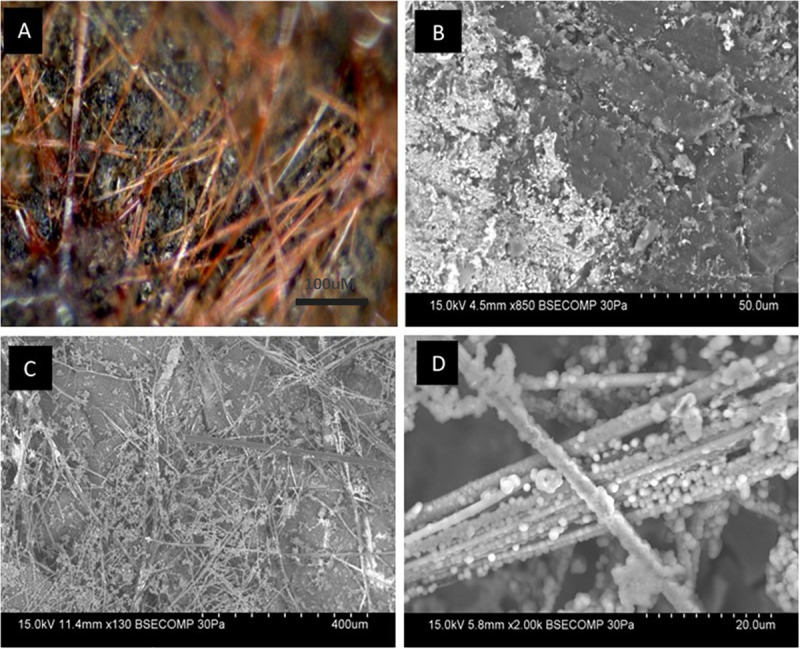
Light and electron microscopy images of neutral red crystals on coal. **(A)** Crystals can be observed as a dense layer of orange-red needles randomly oriented across the coal surface (black). Needles vary in width and length. **(B)** Without neutral red, remnants of lysed biofilms can be seen scattered over the surface of Jharia coal. **(C)** For the first time, high resolution electron microscopy images of the crystalline form of neutral red were obtained through Backscattered electron microscopy. **(D)** Shows the nodulation over the crystalline form of neutral red caused by microbes and mineral deposition.

Coal surfaces incubated in the presence of groundwater with incumbent community and neutral red, were subject to light and scanning electron microscopy. Figure 5A shows neutral red crystals on the coal surface as slender red-orange needles that form a mesh across the surface of the coal; Figure 5B shows electron microscopy images of coal samples inoculated with groundwater containing a coal seam associated community. Without neutral red, cells are scattered with relatively few intact. There is some evidence of debris on the Jharia coal surface which may be remnants of lysed biofilms. Figure 5C shows, for the first time, high resolution electron microscopy images of the crystalline form of neutral red. The image presented were obtained with minimal specimen preparation as ethanol dissolves the crystals that may result in artifacts. The crystals appear to be nodulated in a manner reminiscent of both plant root nodulation caused by microbes and mineral deposition. These data are consistent with the crystalline form of neutral red serving to extract reducing equivalents from microbes or coal or reduced minerals given their co-location at the coal-groundwater interface, and provide another vision of the glassy looking material observed in Figure 5. Figure 5D, shows the nodules at high resolution that may be deposition of inorganic precipitate, a biological formation (cells) or a combination of abiotic and biological component over the neutral red crystals.

**FIGURE 6:**
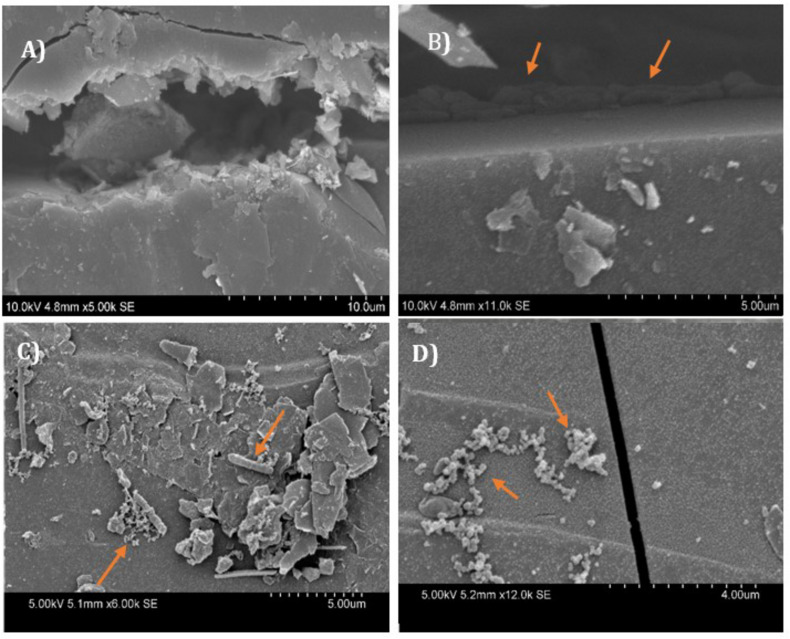
Electron microscopy images of the Jharia coal samples, inoculated with a coal seam associated microbial community in groundwater and treated with neutral red. The HDMS method was used to prepare the coal samples, for the electron microscopy. Image **(A)** is showing a natural fracture at the coal surface. Microbes can also be seen inside these natural fracture **(B)**, and over the surface of coal **(C,D)**. Arrows showing the bacterial colonies/archaea, inside and on the surface of the coal.

Figure 6 shows high-resolution electron microscopy images of microbes on the surface of coal. The samples were prepared using the HDMS method (Hazrin-Chong and Manefield, 2012), which involves washing samples with solvents so neutral red crystals would be dissolved. Figure 6A, represents the natural fracture of the coal, the Figure 6B is showing a colony of rod-shaped bacteria or archaea utilizing the natural fractures of coal as their habitat and also as a source of substrate. The Figures 6C,D are showing different colonies of bacteria or archaea, over the surface of coal.

### Surface Analysis of Coal

Changes in coal surface chemistry caused by groundwater microbes with/without neutral red, was analyzed by ATR-FTIR spectroscopy and XPS. The use of an ATR crystal for sampling provides a depth sensitivity of approximately 3 μm. Changes in the infrared spectrum reflects the formation or depletion of organic functional groups as a result of microbial activity in this surface region. A stackplot of the ATR-FTIR spectra of sub-bituminous coal incubated with groundwater and neutral red, coal incubated with groundwater with no neutral red, coal incubated with ground water and sodium azide (sterile control) and coal only, is shown in Supplementary Figure 1. Previous studies have shown that biogenic oxidation activity on the coal surface, by *Pseudomonas fluorescens* impacts the carbonyl (C = O) stretching region of sub-bituminous coal spectra, occurring around 1700 cm^–1^ (Hazrin-Chong et al., 2014). However, ATR-FTIR of samples treated with the groundwater inoculum used in this study showed no significant difference in carbonyl (C = O) stretching region at 1700 cm^–1^.

**FIGURE 7:**
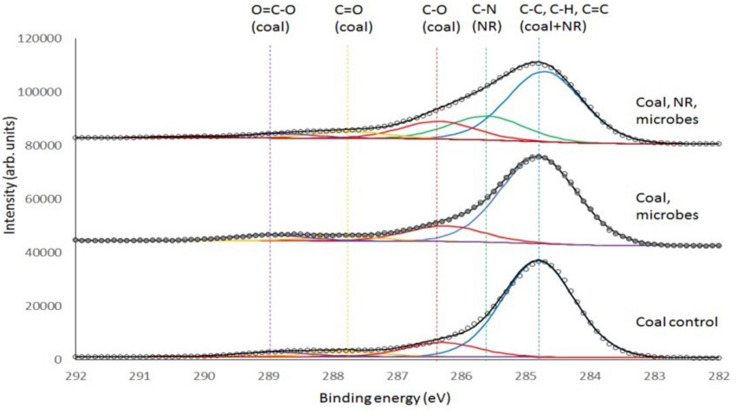
Deconvoluted XPS spectra of Jharia coal as control, Jharia coal with groundwater, and Jharia coal treated with groundwater and neutral red. Carbon chemical state assignments are color coded and generally common between samples with the exception of C-N that is only present in the sample with neutral red (NR).

Consequently, a second analysis of the coal samples with XPS was undertaken (Figure 7). XPS uses a monochromatic X-ray beam to eject photoelectrons from the core shell of an atom and these photoelectrons have a limited escape depth (<5 nm) from the sample providing a much greater degree of surface sensitivity compared to ATR-FTIR. The binding energy of the carbon 1s photoelectron is sensitive to the chemical environment around the carbon atom, providing an alternate method to investigate changes in organic functional groups at the surface of the solid coal sample. Please refer to the Supplementary Figure 3 for the XPS analysis of only ground water (the XPS data of groundwater with neutral red is provided as a separate file).

Figure 7 and Table 1 shows that the coal O = C-O groups on coal increase in the presence of microbes and increase again in the presence of neutral red with microbes.

**TABLE 1:** Ratio of XPS peaks to show the influence of microbes on coal.

**Ratio**	**Coal**	**Coal + Groundwater**	**Coal + NR + Groundwater**	**Comment**
O = CO/(C-C, C-H)	0.047	0.065	0.088	Ratio of “acetate” (as O = C-O) *increases relative to coal C-C, C-H*
C-O/(C-C, C-H)	0.15	0.18	0.36	Ratio of C-O groups *increases*

*(C-C, C-H) signal in (Coal, NR, and Ground water) has been adjusted to remove the NR signal [the carbon 1 s signal from neutral red was removed from the XPS data to produce a carbon spectrum for the coal surface. This was performed by assuming that neutral red was the only source of the nitrogen, so the maximum possible contribution of neutral red to the carbon signal was a factor of 15/4 of the atomic% from the nitrogen 1 s signal, using the molecular formula for neutral red (C_15_H_17_N_4_)].*

## Discussion

Acetogens are strictly anaerobic bacteria and ubiquitous in nature. With methane-forming archaea, they constitute the final step in the production of methane from coal. Acetogens utilize the Wood–Ljungdahl (Ragsdale, 2008) pathway for the production of acetyl-CoA and also for biofuel production such as ethanol, butanol or hexanol. Bio-commodities including acetate, lactate, butyrate, hexanoate, 2,3-butanediol and acetone are produced by acetogens, by using syngas as the carbon and energy source. All acetogens that produce organic acids like acetic, butyric or other acid are described as acetogenic (Schiel-Bengelsdorf and Dürre, 2012) while the acetogens that produce solvents like ethanol, butanol, and hexanol could be described as solventogenic (Bengelsdorf et al., 2018). Acetogens also employ a type of chemolithoautotrophic metabolism and (Müller, 2003) provide capabilities to perform; (a) the oxidation of CO into CO_2_, (b) using reducing equivalents derived from the above reaction for their growth, (c) assimilate the part of CO_2_ formed via ribulose-bisphosphate cycle, and (d) to withstand CO inhibition. A total of 61 strains were described to be acetogens, 56 are reported to grow using H_2_ + CO_2_, three strains are known to use CO, not H_2_ + CO_2_, and the remaining two are included because of having the gene cluster of Wood-Ljungdahl pathway (Bengelsdorf et al., 2018).

The Wood-Ljungdahl pathway has two branches, the methyl and carbonyl branch, with both contributing to the formation of central intermediate acetyl-CoA. During heterotrophic growth, with sugar as energy and carbon source, pyruvate by glycolysis, enters the Wood-Ljungdahl pathway (Bengelsdorf et al., 2018). Ferredoxin oxidoreductase catalyzes and convert pyruvate into acetyl-CoA with the production of CO_2_. Then, this CO_2_ is reduced to produce acetyl-CoA in the Wood–Ljungdahl pathway, with the reducing equivalents produced during the process of glycolysis, however, the methyl branch is involved in the stepwise reduction of produced CO_2_ into methyl group, which is needed to produce acetyl-CoA.

**FIGURE 8:**
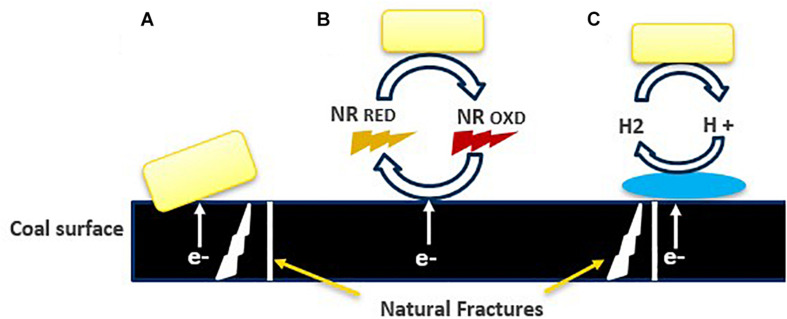
Overview of proposed extracellular electron transfer mechanism (Lovley, 2011): **(A)** direct, **(B)** electron transfer mediated by dissolved redox mediators, and neutral red (proposed hypothesis), indicating that chemical processes occurring at nanoscale, **(C)** dependent on the H_2_ generation catalyzed by extracellular hydrogenase.

A different mechanism of extracellular electron transfer (EET) has been proposed for acetogens (Figure 8; Bengelsdorf et al., 2018). Direct EET involves outer-membrane bound cytochromes, transporting extracellular electrons from outside to inside of the cells and the other way around. This mechanism is well studied in *Geobacter* sp. The species of *Moorella* and *Sporomusa*, have cytochromes but most other acetogens are lacking them. *Shewanella oneidensis* and *Pseudomonas aeruginosa* use flavins and phenazines to mediate the transport of electrons (Bengelsdorf et al., 2018). Another possibility is an indirect electron exchange based on the evolution of H_2_.

During methanogenesis in *Methanosarcina* species, multiple reduction-oxidation reactions take place, mediated partly by a membrane bound phenazine molecule called methanophenazine. In *Methanosarcina mazei*, methanophenazine relays electrons through the cytoplasmic membrane from a membrane bound F_420_H_2_ dehydrogenase and membrane bound heterodisulphide reductase (Deppenmeier, 2004).

Neutral red and methanophenazine are structurally related phenazines with similar mid-point potentials (E^0^ −300 to −400 mV), and Beckmann *et al.*, demonstrated that NR mimics methanophenazine, in the respiratory chain of *Methanosarcina mazei*. In addition, NR forms crystals, at the concentration of 250 μM in anoxic culture media and these crystals accept electrons more efficiently than the soluble form of neutral red. NRCs accept electrons more efficiently because its midpoint potential is 444 mV, more positive at +69, than the soluble neutral red of −375 mV. This makes the crystalline form more amenable to attract electron from organic and inorganic electron carriers present in environment and make them available to the microbes.

Neutral red molecules solubilized in the reduced state by protonation at the point of methanogen cell contact, with the crystal surface deliver electrons to the methanogens at a negative midpoint potential. Reduced neutral red delivers electrons to the heterosulfide reductase (HDrED) in the membrane, resulting in proton translocation and liberation of CoM-SH and CoB-SH enhancing the rate of methanogenesis (Beckmann et al., 2015). With this phenomenon, increased methane production was recorded, but nothing is known about the impact of neutral red on the microbial communities involved in the other stages in the bioconversion of coal to methane.

Light microscopy of coal surfaces, in the presence of the coal-seam groundwater with neutral red indicated that the crystals distribute widely over the surface of coal and have coating on them. The deposition over crystals are cells, and mineral deposition. The backscattered electron microscopy of crystals provided a clearer picture of the crystals. The crystals, because of their midpoint potential, have higher efficiency to attract reducing equivalents from microbes or coal or reduced minerals, and behave like an electron distribution hub for microbes. The microbial communities, involved in different steps of biodegradation of coal, could have accepted the electrons for their growth and metabolism from these crystals (Park et al., 1999; Park and Zeikus, 1999), and have attached themselves on the crystalline surfaces of these crystals. However, the electron microscopy of the coal, treated with groundwater, in the absence of neutral red, revealed limited colonization of the coal surface. Electron microscopy of coal samples, prepared by the HMDS method (Hazrin-Chong and Manefield, 2012) which preserves cell structure, showed different cell morphologies widely distributed over the surface of coal. This also indicates that most of the microbial communities tend to associate with the neutral red crystals and thus take benefit from it, if is this provided in the environment. The HMDS method involves the use of strong solvents that dissolve the crystals which is why no crystals are visible over the coal surface.

The acetate production occurs at the interface where microbes are localized on a coal surface coated with neutral red crystals. The surface analysis demonstrates that the acetate production resulting from the oxidation of the coal which was only found a few nanometers into the coal surface. The surface of coal was analyzed by ATR-FTIR and XPS spectroscopy, to find out the changes caused by coal seam water associated microbial communities with and without neutral red. ATR-FTIR spectroscopy did not show differences between treatments because of the limited depth sensitivity of the ATR-crystal. However, XPS, which provides ca. 5 nm surface sensitivity, showed that acetate O = C-O groups and C-O group on the coal surface increased in the presence of neutral red with microbes. The origin of the O = C-O and C-O signal in the XPS is assumed to be from near-surface microbial oxidation, however, it is possible that there is a contribution from carbonyl-containing biomolecules originating from the organisms. The acetate production indicates that the microbial communities involved in conversion of coal into acetate, react with the very top and exposed surface of the coal, and that this reaction benefited from the presence of neutral red crystals. The XPS analysis of the groundwater sample found to be contain organic matter with C-O, C = O and O = C-O groups which could be associated with dissolved fatty acids and other organic material, possibly derived from humic leachates of soil organic matter, algal extracellular polymeric substances (EPS), and dead microbial matter. The adsorption of this material on the surface would enhance the XPS signals for the coal with microbe spectrum. The XPS data for the coal with microbe experiment cannot be definitively ascribed to microbial oxidation processes at the surface. However, the relative enhancement of the O = C-O and C-O signals in the XPS when neutral red is present can only be explained if the microbes are consuming the coal surface. No measurable oxidation product (acetate) is produced when only neutral red is present in the groundwater (no coal provided). The XPS analysis of NR with groundwater data did not show any acetate and is provided as a separate Supplementary Excel File.

The acetate analysis by GC, indicated the production of acetate, was double in the presence of neutral red, than the cultures without neutral red. The microbial communities involved in acidogenesis and acetogenesis during the conversion of coal into methane, might have taken benefits from NRCs by giving or consuming the reducing equivalents instead of just acetoclastic methanogens (Beckmann et al., 2015) and these electrons may have transported inside the cells through various channels, present in the cell membrane or by following direct interspecies electron exchange (Figure 8B) however, the hypothesis of accepting electrons by acetogens and other microbial communities from NRCs still needs to be proven by redox potential-based analytical methods including cyclic voltammetry.

## Data Availability Statement

The original contributions presented in the study are publicly available. This data can be found in the European Nucleotide Archive, under the accession number PRJEB39572.

## Author Contributions

PS and CM carried out the implementation of the framework with the input from AG and ZJ, were in charge of overall planning and direction, analyzed the data, and wrote the manuscript with the contribution of all other authors. All authors contributed to complete the study.

## Conflict of Interest

The authors declare that the research was conducted in the absence of any commercial or financial relationships that could be construed as a potential conflict of interest.
