# Supplementary material for: Surface Analysis of Coal Indicating Neutral Red Enhances the Precursor Steps of Methanogenesis
Source: Front Microbiol. 2020 Nov 9;11:586917. doi: 10.3389/fmicb.2020.586917 (PMC7680738; doi:10.3389/fmicb.2020.586917)
Supplement: Supplementary file 1 [file Data_Sheet_1.docx]

Supplementary Material

# Supplementary Figures


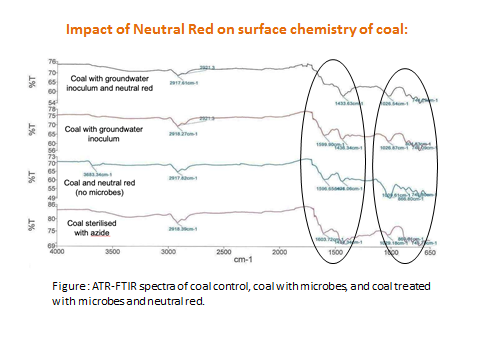


Supplementary Figure 1 ATR-FTIR Spectra of sub-bituminous Jharia coal incubated with groundwater and neutral red. 1 is coal incubated with groundwater with neutral red, 2 is coal incubated with ground water without neutral red, 3 is coal with neutral red, no ground water was provided and; 4 is coal with the ground water and sodium azide (sterile control).


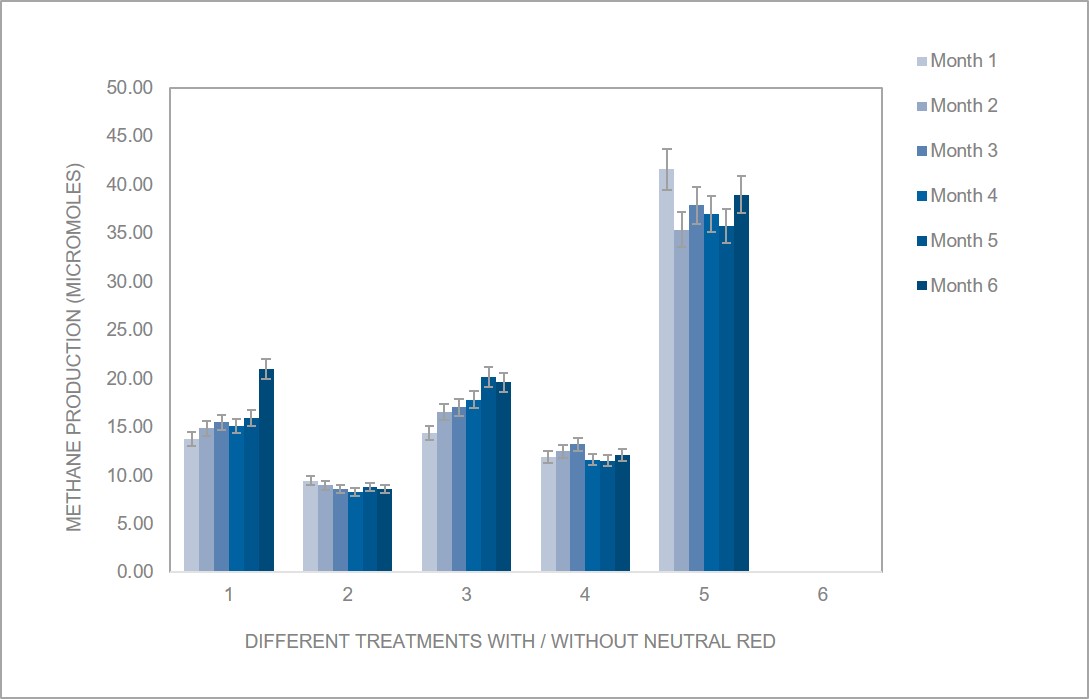

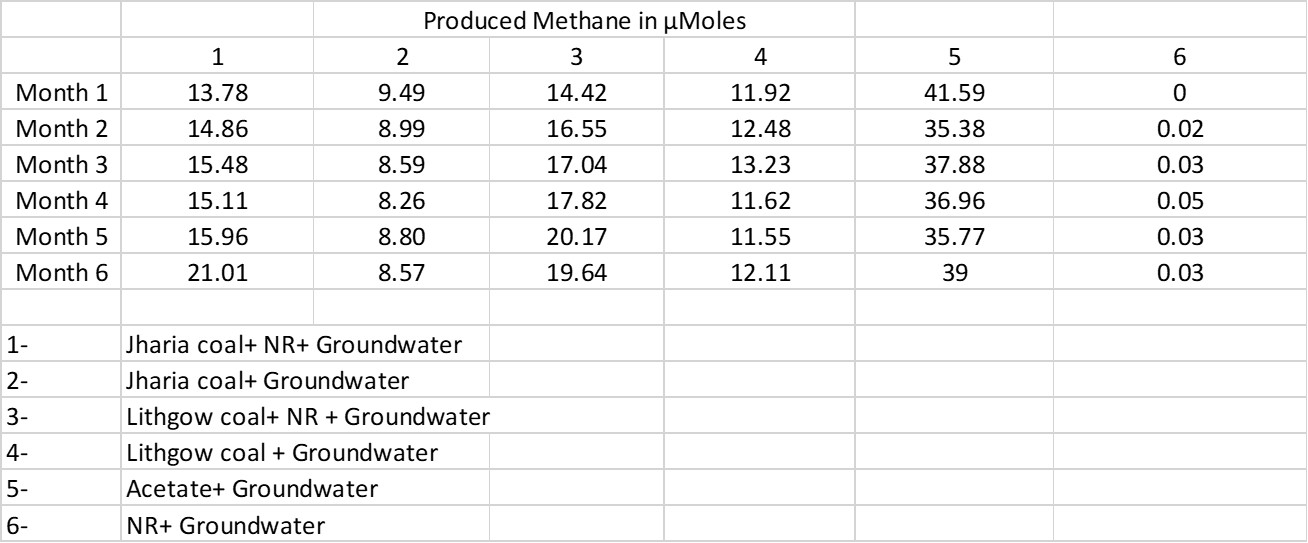


**Supplementary Figure 2**: The amount of methane produced over time in the presence/ absence of 250µM of neutral red. The acetate represents the positive control. The error bars represent the standard errors.


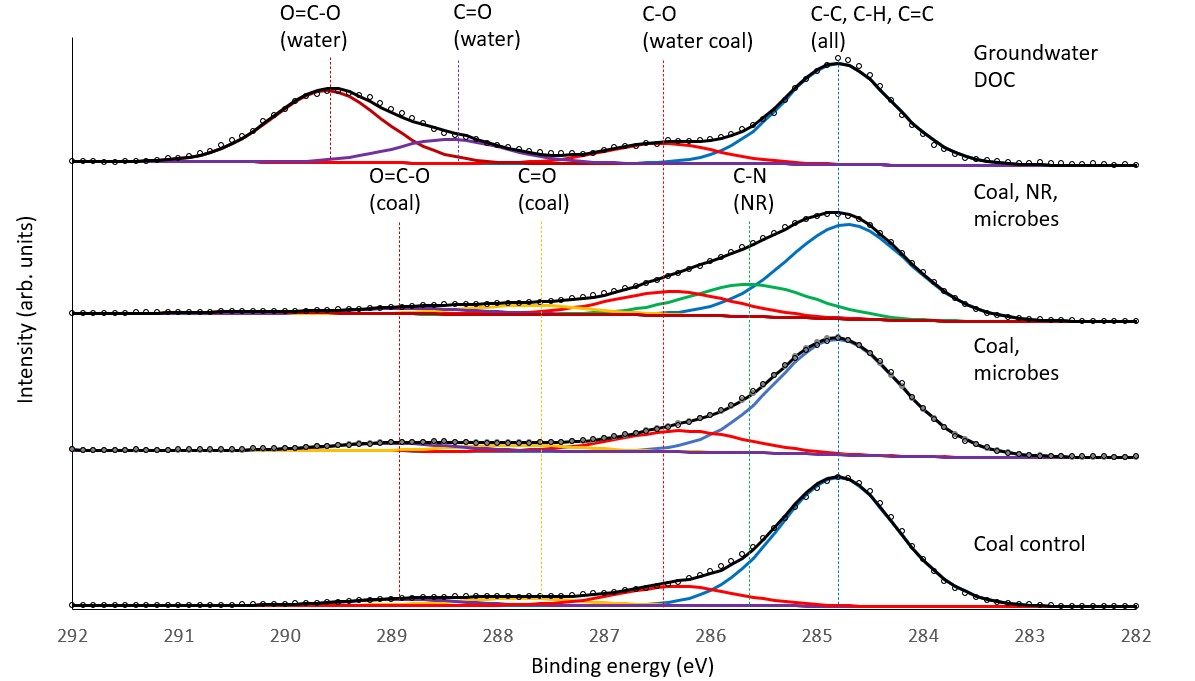


Supplementary Figure 3. Deconvoluted XPS spectra of groundwater as negative control, Jharia coal as control, Jharia coal with groundwater, and Jharia coal treated with groundwater and neutral red. Carbon chemical state assignments are colour coded and generally common between samples with the exception of C-N that is only present in the sample with neutral red (NR). In groundwater XPS spectrum the O=C-O and C=O peaks appear at higher binding energies compared to the coal spectra. This may be due to charging effects on the coal.
